# Work environment, job satisfaction and burnout among Spanish dentists: a cross-sectional study

**DOI:** 10.1186/s12903-021-01480-9

**Published:** 2021-03-24

**Authors:** Javier Molina-Hernández, Lucía Fernández-Estevan, Javier Montero, Lorena González-García

**Affiliations:** 1 Department of Odontology, Faculty of Health Sciences, Universidad Europea de Valencia, 46010 Valencia, Spain; 2 Department of Surgery, Faculty of Medicine, University of Salamanca, Campus Miguel de Unamuno, 36007 Salamanca, Spain; 3 Department of Stomatology, Faculty of Medicine and Dentistry, University of Valencia, 46010 Valencia, Spain; 4 Department of Psychology, Faculty of Health Sciences, Universidad Europea de Valencia, 46010 Valencia, Spain

**Keywords:** Mental health, Health care workers, Dentist, Occupational health practice, Burnout

## Abstract

**Background:**

The main aim of the present study was to examine the relationships among work environment, job satisfaction and burnout in dentists and to analyse the way in which certain sociodemographic variables, such as gender, professional experience and weekly working hours, predict the perception of the work environment.

**Methods:**

A battery of online questionnaires was sent to 3876 dentists officially registered in the triple-province region of Valencia; the battery included the Survey of Organizational Attributes for Dental Care, the Warr–Cook–Wall Overall Job Satisfaction Scale, the Maslach Burnout Inventory and a series of sociodemographic questions formulated for the specific purpose of this study. To assess the relations with the independent variables, we calculated the Pearson correlation coefficient, the Z-scores were calculated to make effect sizes comparable, and the associations between the scales and the sociodemographic variables were investigated by adjusted multiple regression analysis.

**Results:**

A total of 336 participants (9.4%) correctly completed the survey in this study. The mean (M) age was 37.6 years old (standard deviation (SD) = 9.6, median (M_e_) = 34). Participants reported high scores on the work environment and job satisfaction scales, with only limited experiences of burnout (3.8%). Work environment and burnout were significantly and positively predicted by years of professional experience (β = .078; *p* = .000 and β = .107; *p* = .004, respectively), and job satisfaction was significantly and positively predicted by weekly hours of work (β = .022; *p* = .001), without significant differences according to gender.

**Conclusions:**

Dentists who work over 20 hours a week and have more years of professional experience report having better perceptions of well-being at work, with no significant difference according to gender. It is important to highlight the aspects that improve well-being in dentistry to reduce burnout, which would lead to greater work engagement and better attention to patients.

## Background

According to Rugulies [1], the psychosocial work environment is a key research field for understanding how the interrelations of societal structures, environmental exposures, and psychological and psychophysiological processes affect the health and illness of workers, which has potential impacts in terms of wellbeing and discomfort (e.g., job satisfaction, stress, burnout) [2].

Healthcare workers have been shown to be at risk of burnout and low job satisfaction due to the peculiar characteristics of their profession (workload, patient safety, medical errors and work-life balance) [3]. This has generated interest in the study of this particular work environment [4].

Considering the dental context, dentistry has been recognized as a highly stressful profession [5] that has changed considerably over the last decade in terms of socio-occupational aspects. These changes are largely due to the increase in the number of new graduates, as well as to the emergence of new business situations (pluri-employment, franchises, changes in working hours, types of contracts) and the need to become specialized [6].

Job satisfaction refers to a pleasurable or positive emotional state resulting from a job experience [7]. Worker job satisfaction depends on the combination of different factors, such as the expectations of the employee and the demands of the job, as well as skills, social status, communication between colleagues, patients and the work environment [8]. In the dental setting, there are heterogeneous findings regarding job satisfaction and its association with the work environment and burnout. These discrepancies may be explained in part by the use of different measurement instruments [9].

According to Maslach et al. [10], the response to chronic stress at both the personal level and in terms of working relations triggers burnout syndrome, which is characterized by emotional exhaustion, depersonalization and diminished personal accomplishment. Previous literature has shown the prevalence of burnout among dental professionals in European countries to range from 8 to 36% [9, 11]. Previous studies in the Spanish population indicate a dentist burnout prevalence of 13.8–17.2% [12, 13], but these studies did not present any data that correlated this pathology with other psychosocial variables, such as work environment and job satisfaction.

One of the most widely used and validated instruments for assessing the work environment is the Survey of Organizational Attributes in Dental Care (SOADC) [14]. This scale assesses the work environment and other related aspects, such as the quality of care or organization in dental clinics, by using valid and reliable indexes [14], taking into account independent factors such as gender, age and the number of years employed, as suggested by Appelbaum et al. [2].

Some studies in the medical field have examined whether there are any gender differences in the perception of well- or ill-being in work. While some studies argue that there are no gender differences [15, 16], other publications provide evidence that women have poorer perceived job satisfaction than men [2, 17, 18]. Moreover, other studies have shown that men have a worse perception of their work environment and greater psychological problems because women are more often engaged in part-time jobs or because of their lower job expectations [19, 20].

The main objective of the present study was (1) to evaluate the relationships among perceived work environment, job satisfaction and burnout among dentists in the triple-province region of Valencia (Spain) and (2) to test whether the perceived work environment differs between males and females, workers with different professional experience, and workers with different weekly working hours.

## Methods

### Study participants

This cross-sectional study was carried out in a region of Spain (Valencia), and all the participants were required to meet the following criteria: (1) a degree in dental surgery and (2) membership to an official dental association. In Spain, such membership is mandatory to be able to work as a dentist. In the specific case of the triple-province region of Valencia, there were 3876 officially registered dental professionals at the time of the study. The sample was selected using the contact information provided by the professional associations. As we wanted to add all the dentists registered in the official dental association of the triple-province region of Valencia, we did not exclude anyone on the basis of age, gender or specialty. The exclusion criteria were not being a dentist and not being collegiate. Moreover, data from incomplete questionnaires (e.g., questionnaires where the participants did not give consent to participate or left unanswered sections) were deleted due to the impossibility of correctly handling and interpreting the data.

### Study instruments

#### Sociodemographic information

A total of 6 ad hoc questions were presented to obtain information on gender, age, years of professional experience, type of work contract, dental specialization and weekly working hours.

#### Work environment

The satisfaction of dentists with the organizational system within the clinic where they work was explored using the Survey of Organizational Attributes for Dental Care (SOADC) [14]. This scale was adapted to the dental setting and demonstrated adequate internal consistency (α = 0.77) [14]. In our study, we assessed the sociocultural adaptation of the instrument through the Harkness back-translation method [21].

This scale was an adaptation of the Survey of Organizational Attributes for Primary Care (SOAPC) [22], which was created to evaluate the work environment in small primary healthcare practices following the approach of Cohen et al. [23]. The SOACD comprised 21 items divided into four dimensions: (1) communication; (2) decision making; (3) stress/chaos; and (4) changes made. All responses were answered on a five-point scale (1 = strongly disagree to 5 = strongly agree). The value for each dimension and the score for the overall work environment were calculated as the mean score for each dimension and the mean score for the overall work environment, respectively.

#### Job satisfaction

We assessed job satisfaction with the Spanish version of the Warr–Cook–Wall Overall Job Satisfaction Scale (WCW) [24]. This scale includes 15 items that measure two dimensions: intrinsic and extrinsic job satisfaction. The intrinsic factors address aspects such as recognition for work, responsibility, promotion, or aspects related to the content of the task. The extrinsic factors assessed worker satisfaction with aspects related to the organization of work, such as schedules, remuneration, and physical conditions. Each item was answered on a 7-point Likert scale (1 = extreme dissatisfaction to 7 = extreme satisfaction). The value for each dimension and the score for overall job satisfaction were calculated as the mean score for each dimension and the mean score for overall job satisfaction, respectively. The Spanish version demonstrated adequate internal consistency (α = 0.85–0.88) [24].

#### Burnout

To evaluate burnout experienced by the professionals, we used the Spanish version [25] of the Maslach Burnout Inventory (MBI) [26]. This scale assesses work-related strain and the feelings and attitudes of the professionals towards the patients and their work. Scoring was based on a 7-point Likert scale (1 = never to 7 = every day), and three subscales were established: (1) emotional exhaustion preventing professionals from providing their patients with correct and kind care; (2) depersonalization, where professionals experience negative feelings and attitudes towards their patients, causing them to believe that they deserve the problems they have; and (3) diminished personal accomplishment, characterized by a tendency to constantly underrate the personal work done, with feelings of unhappiness and dissatisfaction [26].

The instrument comprises a total of 22 items, and its reliability and validity have been assessed by multiple studies, both in the dental field [27, 28] and in other healthcare areas [29]. The diagnosis of burnout requires high scores on each of the three subscales. The value for each dimension and the score for burnout were calculated as the mean score for each dimension and the mean score for burnout. The following cut-off points were used to define the prevalence corresponding to each subscale in the Spanish population: emotional exhaustion (high < 15, average 15–24, low > 24), depersonalization (high < 4, average 4–9, low > 9) and diminished personal accomplishment (high < 33, average 33–39, low > 39) [12].

### Study procedure

The study was approved by the Ethics Committee of the *Universidad Europea de Madrid* (Madrid, Spain) (registry: CIPI/038/17). Information was collected via a battery of online questionnaires that remained active for 6 months on the Surveymonkey® website (Menlo Park, Palo Alto, CA, USA), and following the instructions of the official colleges of each region, we were only allowed to contact the dentists once. An informed consent form was presented beforehand to explain that participation in the study was voluntary and anonymous and that the participants could abandon the study whenever they wished. Completing this part was mandatory to continue with the questionnaires. The questionnaires were set up so that subjects could only participate once by controlling the IP address and were designed in such a way that answering each question was obligatory before being able to continue to the next page.

### Statistical analysis

Categorical variables are described using frequencies for categorical variables and means and standard deviations for continuous variables. Parametric analysis was conducted because of the large sample size. The internal reliability was assessed using Cronbach’s α. Firstly, Pearson correlations coefficients were estimated between all the study variables. Z-scores for work environment dimensions, overall job satisfaction and burnout were calculated to make effect sizes comparable. Differences in mean Z-scores according to gender, years worked and working hours per week (recoded to categorical intervals) were assessed by Student’s *t *test and one-way ANOVA F-test. Multiple comparison tests were based on Bonferroni’s criteria. To control the effect of confounders, such as age, on the previous results, multiple regression models were used. Each dependent variable (dimensions of work environment, job satisfaction and burnout) was related to all independent variables (working hours per week, years worked and age) according to a stepwise model for their introduction into the model. Beta coefficients and 95% confidence intervals were estimated, and the R^2^ coefficient was used to assess the goodness of fit. Statistical significance was indicated by a *p* value ≤ 0.05. SPSS 21.0 (IBM SPSS Statistics, Chicago, IL, USA) was used for the analysis.

## Results

The questionnaires were delivered to a total of 3876 dentists. Of these, 557 (14.3%) completed the survey, but only 366 completed it correctly (9.4%).

The age of the participants was between 24 and 69 years old (M = 37.6, SD = 9.6, M_e_ = 34), and 71% were women. A total of 35.2% of respondents worked more than 40 hours a week, while only 6.3% worked less than 20 hours. The dentists had been working for an average of 13 years (SD = 8.7, M_e_ = 11). With regard to professional specialization, most of them (42.1%) worked as general dentists, while 26.2% worked in surgery and periodontology, 17.3% worked in orthodontics/odontopaediatrics, and 14.5% worked in endodontics. The great majority (83.8%) worked in a private practice, with 36.6% working in their own clinic and 30.3% in other clinics. In turn, 17.2% combined work in their own clinic with work in other clinics, and only 16% were contracted staff.

The Survey of Organizational Attributes for Dental Care (α = 0.76 – 0.88) and the Maslach Burnout Inventory (α = 0.74) showed adequate internal consistency, and the Overall Job Satisfaction Scale showed excellent internal consistency (α = 0.93).

The results in Table 1 show that 96.8% of the participants yielded scores above three points on a five-point scale for all dimensions of the work environment, except the dimension of stress (M = 2.87, SD = 0.79). A total of 96.3% of the participants yielded scores above three points on a seven-point scale for all dimensions of job satisfaction (M = 5.07, SD = 1.07). With regard to burnout, 26.2% of the dentists participating in the study yielded scores under three points on the seven-point scale (M = 2.64, SD = 0.85), and 96.2% did not experience burnout (Table 2).

**Table 1:** Distribution of the variables studied according to several score ranges

Scale	Range	M	SD							
SAODC	1–5					1-Strongly disagree	2-Disagree	3-Neutral	4-Agree	5-Strongly agree
Communication	1–5	3.58	.43			0%	0.5%	14.2%	77.6%	7.7%
Decision making	1–5	3.44	.54			0.8%	1.9%	20.2%	68.3%	8.3%
History of change	1–5	3.40	.76			1.1%	5.5%	30.3%	50.5%	12.6%
Overall work environment	1–5	3.30	.31			2.2%	1.1%	84.2%	12.6%	0%
WCW	1–7			1-Extremely dissatisfied	2-Very dissatisfied	3-Moderately dissatisfied	4-Not sure	5-Moderately satisfied	6-Very satisfied	7-Extremely satisfied
Intrinsic job satisfaction	1–7	5.37	1.09	0%	0.5%	2.5%	11.5%	19.4%	37.4%	28.7%
Extrinsic job satisfaction	1–7	5.00	1.11	0%	0.8%	4.6%	15.8%	26.5%	35%	17.2%
Overall Job satisfaction	1.7	5.17	1.07	0.3%	3.4%	4.1%	10.4%	21%	41.1%	19.7%
MBI				1-Never	2-A few times a year or less	3-Once a month or less	4-A few times a month	5-Once a week	6-A few times a week	7-Every day
Emotional exhaustion	1–7	3.69	1.31	0%	12%	21.6%	28.4%	19.7%	13.4%	4.9%
Depersonalization	1–7	2.11	1.02	0%	0%	13.7%	24.9%	14.2%	1.6%	0.3%
Diminished personal accomplishment	1–7	1.76	.77	18.3%	51.6%	23%	5.7%	1.1%	0%	0%
Burnout	1–7	2.64	.85	0%	26.2%	43.4%	23.5%	6.3%	0.3%	0.3%

**Table 2:** Distribution of burnout dimensions according to score ranges to estimate the prevalence of burnout syndrome in the tested population

Dimension	Score	N	%
Emotional exhaustion	Low (< 15)	78	21.3
	Medium (15–24)	121	33.1
	High (< 24)	167	45.6
Depersonalization	Low (< 4)	165	45.2
	Medium (4–9)	125	34.2
	High (> 9)	75	20.5
Diminished personal accomplishment	Low (< 33)	252	69.0
	Medium (33–39)	82	22.5
	High (> 39)	31	8.5
Burnout	Yes	14	3.8
	No	352	96.2

**Fig. 1:**
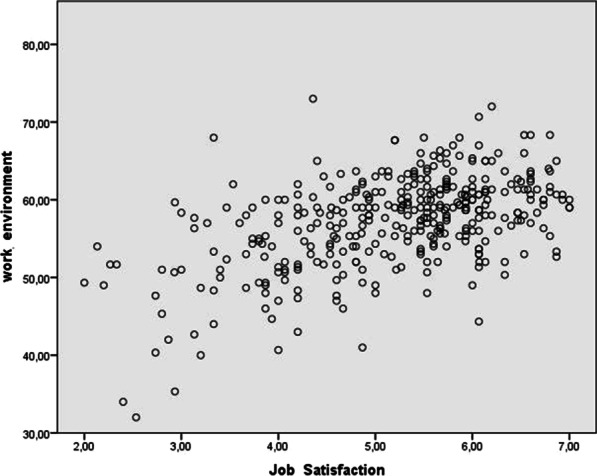
Graphic representation of the association between job satisfaction and work environment

**Fig. 2:**
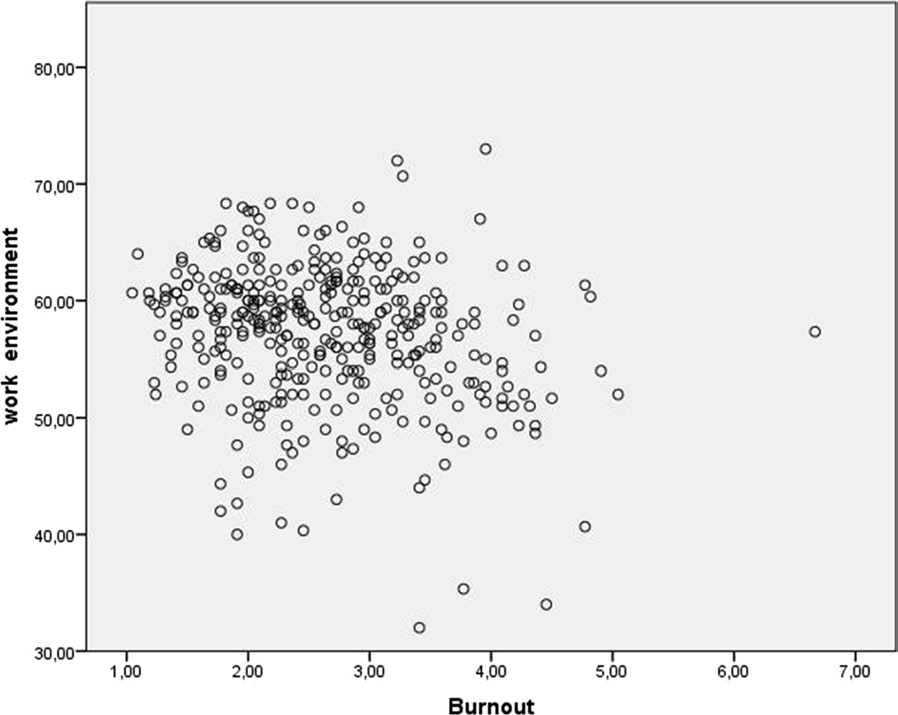
Graphic representation of the association between burnout and work environment

Pearson correlation coefficient analysis showed a significant and positive association between work environment and overall job satisfaction (*r* = 0.50; *p* < 0.001) (see Table 3 and Fig. 1). Work environment significantly and negatively correlated with burnout (*r* = − 0.18; *p* < 0.001) (see Fig. 2) and with all burnout dimensions (diminished personal accomplishment (*r* = − 0.20; *p* < 0.001). emotional exhaustion (*r* = − 0.13; *p* < 0.05) and depersonalization (*r* = − 0.13; *p* < 0.05) (Table 3).

**Table 3:** Associations between the study variables: Pearson correlations coefficients (*r)*

Vaiable	1	2	3	4	5	6	7	8	9	10	11	12
1. Emotional exhaustion												
2. Depersonalization	.416^**^											
3. Diminished personal accomplishment	.347^**^	.407^**^										
4. Burnout	.880^**^	.691^**^	.679^**^									
5. Communication	− .159^**^	− .179^**^	− .252^**^	− .240^**^								
6. Decision making	− .138^**^	− .129^*^	− .246^**^	− .203^**^	.502^**^							
7. Stress/chaos	.484^**^	.336^**^	.305^**^	.507^**^	− .256^**^	− .413^**^						
8. History of change	− .046	.048	− .010	− .021	.269^**^	.355^**^	− .086					
9. Overall work environment	− .131^**^	− .135^*^	− .204^**^	− .187^**^	.646^**^	.879^**^	− .310^**^	.601^**^				
10. Intrinsic job satisfaction	− .405^**^	− .297^**^	− .455^**^	− .482^**^	.394^**^	.492^**^	− .559^**^	.194^**^	.464^**^			
11. Extrinsic job satisfaction	− .477^**^	− .304^**^	− .378^**^	− .507^**^	.444^**^	.542^**^	− .602^**^	.219^**^	.518^**^	.874^**^		
12. Overall job satisfaction	− .458^**^	− .310^**^	− .428^**^	− .512^**^	.435^**^	.537^**^	− .601^**^	.214^**^	.509^**^	.936^**^	.972^**^	

^**^Significant association (*p* < 0.01, two-tailed); *Significant association (*p* < 0.05, two-tailed)

Burnout significantly and positively correlated with the dimension of stress (*r* = 0.50; *p* < 0.001) and negatively correlated with overall job satisfaction (*r* = − 0.51; *p* < 0.001), the dimension of communication (*r* = − 0.24; *p* < 0.001) and decision making (*r* = − 0.20; *p* < 0.001) (Table 3).

Table 4 shows the differences in mean Z-scores for work environment according to the selected sociodemographic variables. Males obtained significantly greater scores in the dimensions of communication, decision making and changes and less stress. Males had a significantly better perception of the overall work environment than females (0.10 ± 1.01 versus − 0.04 ± 0.99, respectively). In addition, the perception of the overall work environment was seen to improve with the years of professional experience and the number of hours worked per week.

**Table 4:** Differences in mean work environment Z-scores according to sociodemographic variables

Variable	Communication	Decision making	Stress/chaos	History of change	Overall work environment
Gender					
Male	0.18 ± 0.96	0.16 ± 0.96	− 0.16 ± 0.98	0.20 ± 0.92	0.10 ± 1.01
Female	− 0.07 ± 1.01	− 0.06 ± 1.03	0.07 ± 1.00	− 0.08 ± 1.02	− 0.04 ± 0.99
	*p* = 0.029*	*p* = 0.066	*p* = 0.045*	*p* = 0.013*	*p* = 0.004**
Years worked					
< 5	− 0.37 ± 1.09^a^	− 0.31 ± 1.37^a^	0.14 ± 1.04^a^	− 0.37 ± 1.00^a^	− 0.33 ± 1.25^a^
6–10	0.16 ± 1.00^b^	− 0.08 ± 0.88^ab^	0.24 ± 1.04^a^	0.08 ± 0.91^b^	0.19 ± 1.03^b^
11–20	0.02 ± 0.87^ab^	0.12 ± 0.96^ab^	− 0.06 ± 0.82^ab^	0.06 ± 1.01^ab^	0.06 ± 0.94^ab^
> 20	0.12 ± 1.06^b^	0.24 ± 0.80^b^	− 0.40 ± 0.96^b^	0.23 ± 1.00^b^	− 0.02 ± 0.84^ab^
	*p* = 0.010*	*p* = 0.009**	*p* < 0.001***	*p* = 0.006**	*p* = 0.018*
Working hours per week					
< 20 h	− 0.77 ± 0.86	− 0.66 ± 1.07^a^	0.02 ± 1.07^a^	− 0.30 ± 0.99^a^	− 0.73 ± 1.03^a^
20–30 h	− 0.07 ± 1.00^a^	− 0.03 ± 1.03^ab^	− 0.20 ± 0.98^a^	− 0.18 ± 0.92^a^	− 0.25 ± 0.91^ab^
30–40 h	0.05 ± 0.99^a^	0.10 ± 0.91^b^	− 0.02 ± 0.96^a^	0.06 ± 1.01^a^	0.08 ± 0.93^bc^
> 40 h	0.13 ± 0.98^a^	0.04 ± 1.07^b^	0.14 ± 1.03^a^	0.10 ± 1.02^a^	0.19 ± 1.04^c^
	*p* = 0.001**	*p* = 0.010*	*p* = 0.124	*p* = 0.098	*p* < 0.001***

Independent samples Student’s *t* test was used for gender, and one-way ANOVA F-test was used for years worked and working hours per week. The same superscript letter means that there were no differences between means according to Bonferroni´s multiple comparisons

***Significant association (*p* < 0.001, two-tailed); **Significant association (*p* < 0.01, two-tailed); *Significant association (*p* < 0.05, two-tailed)

To control the effect of confounding factors, multiple regression models were used, as shown in Table 5. The results indicate that gender did not predict the overall work environment. The multiple model showed that the results were strongly confounded by age for the dimension of stress (β = − 0.020; *p* < 0.001), explaining 6.2% of the variance (R^2^ = 0.062, *p* < 0.001).

**Table 5:** Multiple regression analysis for different outcomes (dimensions of work environment, job satisfaction and burnout) according to independent variables (working hours per week, years worked and age)

	Beta	95% CI	*p* value	R^2^ (%)
Model 1. Communication				
Working hours per week	0.079	0.027–0.131	0.003**	2.8
Model 2. Decision making				
Years worked	0.012	0.005–0.019	0.001**	3.7
Model 3. Stress/chaos				
Age	− 0.020	− 0.029 to − 0.011	< 0.001***	6.2
Model 4. History of change				
Working hours per week	0.125	0.034–0.216	0.007**	3.7
Age	0.010	0.001–0.018	0.025*	
Model 5. Overall work environment				
Working hours per week	0.078	0.040–0.117	< 0.001***	4.9
Model 6. Overall job satisfaction				
Years worked	0.022	0.009–0.035	0.001**	3.5
Model 7. Burnout				
Working hours per week	0.107	0.004–0.210	0.042*	1.3

The stepwise method was used to enter significant independent variables into the model. Beta coefficients and 95% confidence intervals, *p *value and R^2^ of the model

^***^Significant association (*p* < 0.001, two-tailed); **Significant association (*p* < 0.01, two-tailed); *Significant association (*p* < 0.05, two-tailed)

The number of years worked significantly and positively predicted higher scores on the dimension of decision making (β = 0.012; *p* = 0.001) and job satisfaction (β = 0.022; *p* = 0.001). This variable explains 3.7% of the variance in the dimension of decision making (R^2^ = 0.037, *p* < 0.001) and 3.5% of the variance in job satisfaction perceived by workers (R^2^ = 0.035, *p* < 0.001).

Furthermore, the number of working hours per week significantly and positively predicted the dimension of communication (β = 0.078; *p* = 0.003), history of changes (β = 0.123; *p* = 0.009), overall work environment (β = 0.078; *p* = 0.000), and burnout (β = 0.107; *p* = 0.004). The variable of working hours explained 4.9% (R^2^ = 0.049, *p* < 0.001) of the variance in the overall work environment and 2.2% (R^2^ = 0.02, *p* < 0.05) of the variance in burnout syndrome.

In our study, there is a representative sample of dental professionals in our community, where it has been verified that only 3.8% had burnout. It has been shown how the work environment and the job satisfaction are positively and significantly correlated, as well as that burnout is negatively and significantly correlated to these two variables. Although the data indicated that gender positively predicted the perception of work environment, after the multiple regression analysis it was shown that as the years worked and working hours per week increased, the perception of the work environment and the job satisfaction were higher. This situation is due to a better communication and a lower perception of stress that is achieved over the years, without having any impact gender on the dependent variables.

## Discussion

The present study explored the relationships among work environment, job satisfaction and burnout in dentists and showed differences in the perception of the work environment according to gender, years of professional experience and weekly hours of work.

Regarding our first objective, the great majority of the surveyed dentists (96%) had a good perception of their work environment and job satisfaction, with only 3.8% having experienced burnout.

Focusing on burnout experiences, the prevalence of burnout in the present study was lower than that in other Spanish studies involving periodontists (13.6%). This difference could be because periodontists are involved in periodontal and implant surgery, which can increase stress, leading to higher levels of emotional exhaustion [12]. Our results also differ from those obtained in other European studies, such as that published by Puriene in Lithuania [9], where 83.6% of the sample had experienced burnout, and the study carried out by Collins in Great Britain, where 87.7% of the sample had experienced burnout [5]. These marked differences in burnout level may be explained by the fact that all dentistry specialities were included, which would be interesting for analysis in future studies.

The results of the association between variables (see Table 3) show a significant and positive association between work environment and job satisfaction (*r* = 0.50; *p* < 0.001), which is consistent with the findings of other authors, such as Appelbaum et al. and Hayes et al., in the medical setting (*r* = 0.70; *p* < 0.001) [2, 15]. Wargo et al. [30] observed that work environment alone explained 55% of the variance in job satisfaction and found a positive correlation between both items (*r* = 0.76; *p* < 0.001).

A significant negative association was observed between work environment and burnout (*r* = − 0.18; *p* < 0.001). Similar results have been obtained in other studies conducted in the medical setting [15, 31], despite the use of different questionnaires to analyse work environment (e.g., emotional exhaustion [*r* = − 0.41; *p* < 0.001], depersonalization [*r* = − 0.19; *p* < 0.001] and personal accomplishment [*r* = 0.35; *p* < 0.001]) [15]. Accordingly, the better the work environment, the less common burnout is among professionals. As shown in Fig. 2, the experience of burnout can vary independently of the perceived work environment. This can explain the rates of burnout, although 84.2% of the participants reported perceiving a neutral work environment. This is consistent with the observations of Gorter [32], who underscored that apart from the work environment, the interaction between the individual and the work environment is very important.

On the other hand, burnout showed a significant and negative correlation with overall job satisfaction (*r* = − 0.51; *p* < 0.001) and with two of the dimensions of the work environment: communication (*r* = − 0.24; *p* < 0.001) and decision making (*r* = − 0.20; *p* < 0.001). As seen in Table 2, over 75% of the study sample answered agree or strongly agree on items related to communication (85.3%) and decision making (76.6%) in their clinic, and moreover, close to 60% of them experienced no stress or chaos in the workplace, which could explain the low rate of burnout obtained in our study. These results are consistent with the observations of some studies in the dental practice setting, where certain factors related to the work environment, such as autonomy at work, cooperation at work and staff problems, were negatively associated with burnout [33, 34].

As described by Denton et al. [11]. The positive scores referred to job satisfaction and work environment, and the negative scores referred to burnout, which may be explained by the way in which flexible management contributes positively to the work environment. The dentists surveyed in the present study mostly worked in private practice (83.8%), and 36% were the owners of a dental clinic. This allowed them to tailor the work environment to their own preferences, choosing working hours, staff and material.

In relation to our second objective, there were some individual characteristics associated with the perception of the work environment. Regarding gender, females perceived greater stress and had a poorer perception of their work environment than males, which is in accordance with the findings of previous studies [34]. However, the results of the regression analysis showed that gender did not predict any construct in the study. This is in accordance with the findings of Mottaz, who observed that there is a general tendency in the literature to demonstrate gender differences in the workplace, but there could be other factors involved that are not necessarily linked to gender and can vary according to the situation [35]. Perhaps other differences in lifestyle, such as family situation, pregnancy, and work-life balance as well as their contribution to well-being in dentists, could be further investigated.

As seen in Table 5, there is a clear relationship between age and the level of stress (R^2^ = 0.062, *p* < 0.001), but gender has been shown to be a confounding variable because men have been shown to have a lower perception of stress. These data may come from a baseline bias because they coincide with the fact that the men in our study sample are older and had more years of work experience than women. This may be because medical careers have historically been studied by men, but in recent years, the number of women in these professions has increased to higher degrees and thus has gradually changed the demographic profile [36].

With regard to the years of professional experience, the perception of stress was seen to decrease as the years of experience increased, while all the other dimensions of the work environment improved. Much of the literature has identified the years worked as an important factor associated with well-being, and some studies in the dental field about self-perceived mental health complaints found a positive correlation between professional experience and mental health [9, 37, 38]. However, a study carried out with Spanish dentists did not find statistically significant differences in the burnout score in dentists of different ages [39].

In our study, dentists who were working for more than 20 years had significantly lower perceived stress than those with experience in the shorter ranges (− 0.40 ± 0.96 vs 0.14 ± 1.04). Though the results of the multiple regression analysis showed a clear association of professional experience with the perception of stress and job satisfaction, we are not able to predict both of them with only this variable because of its low predictive value. This relationship between professional experience and well-being could possibly be attributable to greater resilience among these individuals, since past experiences allow them to deal with stress and develop coping skills [40] or to the work engagement among older workers [38].

The variable “working hours” has shown the largest association with burnout and the dimensions of changes made, communication and the overall work environment. However, due to its low predictive value, it would be a mistake to assume that only the number of hours worked per week would predict all these items. Te Brake et al. [16] found that the level of depersonalization in men increases according to the number of working hours per week. However, one study carried out with different health professionals did not find any relationship between working hours and any variable at work [41]. Therefore, further studies are required to verify the relationship between working hours and well-being at work.

To the best of our knowledge, this is the first study in Spain to specifically address the work environment using a validated questionnaire in the sociocultural context of Spanish dentists. From a practical point of view, the SAODC offers the dental care community an adequate tool for measuring the work environment at the national level and can provide visibility to the social and working conditions in dentistry, which is considered a high-risk profession [42]. Additionally, with the objective of supporting and improving our scientific results about the current social and working situation in dentistry, it could facilitate the conduction of new standardized studies.

Furthermore, our results indicated a relationship of burnout and job satisfaction with the sociodemographic aspects of the dentist. This relationship has practical relevance in prioritizing those aspects that will achieve an effective work team (communication, decision making, number of working hours) with a better work environment and hence higher satisfaction at work and a lower degree of burnout. This well-being situation at work, as we can see in the literature in the medical field, would be related to more work engagement [33, 43], less intention to leave [44], better attention to the patients [45] and less turnover [46, 47].

Our study has some limitations to be considered. First, it is important to be aware of the predictive limitations of cross-sectional studies because causal relationships cannot be identified by multiple regression analysis alone; therefore, to clarify causal relationships, it will be necessary to carry out additional prospective research. Second, the level of participation reflected by the questionnaire response rate was low (9.4%), but these data are in concordance with Kelley et al. [48], who said that the response in this type of method is low, near 20%, depending on the content and length of the questionna°ire, especially in the medical field [49]. In this case, we used a long questionnaire, and perhaps professionals with more stress and lower wellbeing may not have been particularly inclined to participate or may not have had enough time to do so. Third, elderly individuals might not have been recruited because of the online nature of the questionnaires [49], and forth, we were only allowed to send the questionnaire one time through the official colleges.

Although the response rate was low, the number of questionnaires completed by the participants in this study (366) was similar to that in other studies in the dental field [16, 50, 51]. However, we understood that this work has an exploratory objective, and the results need to be confirmed with further studies including more participants.

## Conclusion

Based on the results obtained, we consider it important for dentists to have a good perception of their work environment, as this will help to reduce experiences of burnout and thus improve job satisfaction. Furthermore, it may be affirmed that dentists who work over 20 hours a week and who have more years of professional experience will have a better perception of their well-being at work, with no significant differences between genders. It is important to highlight those aspects that improve work-related well-being in dentists to reduce burnout to achieve greater work engagement and better attention to patients. These results are useful to develop more studies and create health promotion programmes for dentists and help them maintain a good quality of life and mental health at work.

## Data Availability

The datasets used and/or analysed during the current study are available from the corresponding author on reasonable request.

## Abbreviations

SOADC: Survey of Organizational Attributes for Dental Care.
WCW: Warr–Cook–Wall overall job satisfaction scale.
MBI: Maslach Burnout Inventory.
M: Mean
SD: Standard deviation
Me: Median

## References

[1] Rugulies R (2019). What is a psychosocial work environment?. Scand J Work Environ Health.

[2] Appelbaum NP, Lee N, Amendola M, Dodson K, Kaplan B (2019). Surgical resident burnout and job satisfaction: the role of workplace climate and perceived support. J Surg Res.

[3] Aalto AM, Heponiemi T, Josefsson K, Arffman M, Elovainio M (2018). Social relationships in physicians’ work moderate relationship between workload and wellbeing-9-year follow-up study. Eur J Public Health.

[4] Piko BF (2006). Burnout, role conflict, job satisfaction and psychosocial health among Hungarian health care staff: a questionnaire survey. Int J Nurs Stud.

[5] Collin V, Toon M, O’selmo E, Reynolds L, Whitehead P (2019). A survey of stress, burnout and well-being in UK dentists. Br Dent J.

[6] Pinilla DJ (2012). Futuro incierto de la profesión de dentista en España. Gac Sanit.

[7] Henne D, Locke EA (1985). Job dissatisfaction: what are the consequences?. Int J Psychol.

[8] Shimizu T, Feng Q, Nagata S (2005). Relationship between turnover and burnout among Japanese hospital nurses. J Occup Health.

[9] Puriene A, Aleksejuniene J, Petrauskiene J, Balciuniene I, Janulyte V (2008). Self-perceived mental health and job satisfaction among Lithuanian dentists. Ind Health.

[10] Leiter MP, Maslach C (2009). Nurse turnover: the mediating role of burnout. J Nurs Manag.

[11] Denton DA, Newton JT, Bower EJ (2008). Occupational burnout and work engagement: a national survey of dentists in the United Kingdom. Br Dent J.

[12] Rios-Santos J, Reyes-Torres M, Lopez-Jimenez A, Morillo-Velazquez JM, Bullón P (2010). Burnout and depression among Spanish periodontology practitioners. Med Oral Patol Oral Cir Bucal.

[13] Falgueras MV, Muñoz CC, Pernas FO, Sureda JC, López MPG, Miralles JD (2015). Burnout y trabajo en equipo en los profesionales de atención primaria. Aten Primaria.

[14] Goetz K, Hasse P, Szecsenyi J, Campbell SM (2016). Questionnaire for measuring organisational attributes in dental-care practices: psychometric properties and test–retest reliability. Int Dent J.

[15] Hayes B, Douglas C, Bonner A (2015). Work environment, job satisfaction, stress and burnout among haemodialysis nurses. J Nurs Manag.

[16] Te Brake H, Bloemendal E, Hoogstraten J (2003). Gender differences in burnout among Dutch dentists. Community Dent Oral Epidemiol.

[17] Gómez-Baya D, Lucia-Casademunt AM, Salinas-Pérez JA (2018). Gender differences in psychological well-being and health problems among European health professionals: analysis of psychological basic needs and job satisfaction. Int J Environ Res Public Health.

[18] Marti KC, Lanzon J, Edwards SP, Inglehart MR (2017). Career and professional satisfaction of oral and maxillofacial surgery residents, academic surgeons, and private practitioners: does gender matter?. J Dent Educ.

[19] Stansfeld S, Candy B (2006). Psychosocial work environment and mental health—a meta-analytic review. Scand J Work Environ Health.

[20] Miao Y, Li L, Bian Y (2017). Gender differences in job quality and job satisfaction among doctors in rural western China. BMC Health Serv Res.

[21] Harkness J, Harkness JA, Van de Vijver FJR, Mohler PP (2003). Questionnaire translation. Cross-cultural survey methods.

[22] Ohman-Strickland PA, John Orzano A, Nutting PA, Perry Dickinson W, Scott-Cawiezell J, Hahn K (2006). Measuring organizational attributes of primary care practices: development of a new instrument. Health Serv Res.

[23] Cohen D, McDaniel RR, Crabtree BF, Ruhe MC, Weyer SM, Tallia A (2004). A practice change model for quality improvement in primary care practice. J Healthc Manag.

[24] Pérez J, Fidalgo M. NTP 394: satisfacción laboral: escala general de satisfacción. Insht; 1993. p. 6. http://www.insht.es/InshtWeb/Contenidos/Documentacion/FichasTecnicas/NTP/Ficheros/301a400/ntp_394.pdf.

[25] Seisdedos N. MBI. Inventario “Burnout” de Maslach: manual; 1997.

[26] Maslach C, Jackson S, Leiter M. Malasch burnout inventory (3rd edition). In: Evaluating stress: a book of resources, vol 1; 1986. p. 191–218.

[27] Hakanen JJ, Perhoniemi R, Bakker AB (2014). Crossover of exhaustion between dentists and dental nurses. Stress Health.

[28] Ahola K, Hakanen J (2007). Job strain, burnout, and depressive symptoms: a prospective study among dentists. J Affect Disord.

[29] Hakanen JJ, Schaufeli WB, Ahola K (2008). The job demands-resources model: a three-year cross-lagged study of burnout, depression, commitment, and work engagement. Work Stress.

[30] Wargo-Sugleris M, Robbins W, Lane CJ, Phillips LR (2018). Job satisfaction, work environment and successful ageing: determinants of delaying retirement among acute care nurses. J Adv Nurs.

[31] Carneiro Monteiro GM, Baeza FLC, Hauck S (2020). Work Environment Evaluation Instrument (WEEI): development, validation, and association with burnout. Trends Psychiatry Psychother.

[32] Gorter RC, Albrecht G, Hoogstraten J, Eijkman MAJ (1998). Work place characteristics, work stress and burnout among Dutch dentists. Eur J Oral Sci.

[33] Gorter RC, Jacobs BLTH, Allard RHB (2012). Low burnout risk and high engagement levels among oral and maxillofacial surgeons. Eur J Oral Sci.

[34] Berthelsen H, Westerlund H, Hakanen JJ, Kristensen TS (2017). It is not just about occupation, but also about where you work. Community Dent Oral Epidemiol.

[35] Mottaz C (1986). Gender differences in work satisfaction, work-related rewards and values, and the determinants of work satisfaction. Hum Relat.

[36] Jefferson L, Bloor K, Maynard A (2015). Women in medicine: historical perspectives and recent trends. Br Med Bull.

[37] Lee CY, Wu JH, Du JK (2019). Work stress and occupational burnout among dental staff in a medical center. J Dent Sci.

[38] Te Brake H, Bouman A-M, Gorter R, Hoogstraten J, Eijkman M (2007). Professional burnout and work engagement among dentists. Eur J Oral Sci.

[39] Varela-Centelles PI, Fontao Valcárcel LF, Martínez González AM, Pita Babío A, Valín Liz MC (2005). Desgaste profesional entre los odontólogos y estomatólogos del Servicio Gallego de Salud. Aten Primaria.

[40] Rada RE, Johnson-Leong C (2004). Stress, burnout, anxiety and depression among dentists. J Am Dent Assoc.

[41] Okazaki E, Nishi D, Susukida R, Inoue A, Shimazu A, Tsutsumi A (2019). Association between working hours, work engagement, and work productivity in employees: a cross-sectional study of the Japanese study of health, occupation, and psychosocial factors relates equity. J Occup Health.

[42] Vodanović M, Sović S, Galić I (2016). Occupational health problems among dentists in Croatia. Acta Stomatol Croat.

[43] Calvo JM, Kwatra J, Yansane A, Tokede O, Gorter RC, Kalenderian E (2017). Burnout and work engagement among US dentists. J Patient Saf.

[44] Sasso L, Bagnasco A, Catania G, Zanini M, Aleo G, Watson R (2019). Push and pull factors of nurses’ intention to leave. J Nurs Manag.

[45] Bai J (2016). Does job satisfaction mediate the relationship between healthy work environment and care quality?. Nurs Crit Care.

[46] Felton JS (1998). Burnout as a clinical entity—its importance in health care workers. Occup Med (Chic Ill).

[47] Goetz K, Campbell S, Broge B, Brodowski M, Steinhaeuser J, Wensing M (2013). Job satisfaction of practice assistants in general practice in Germany: an observational study. Fam Pract.

[48] Kelley K, Clark B, Brown V, Sitzia J (2003). Good practice in the conduct and reporting of survey research. Int J Qual Health Care.

[49] Aitken C, Power R, Dwyer R (2008). A very low response rate in an on-line survey of medical practitioners. Aust N Z J Public Health.

[50] Song KW, Choi WS, Jee HJ, Yuh CS, Kim YK, Kim L (2017). Correlation of occupational stress with depression, anxiety, and sleep in Korean dentists: cross-sectional study. BMC Psychiatry.

[51] Roth SF, Heo G, Varnhagen C, Glover KE, Major PW (2003). Job satisfaction among Canadian orthodontists. Am J Orthod Dentofac Orthop.

